# An altered glial phenotype in the NL3^R451C^ mouse model of autism

**DOI:** 10.1038/s41598-020-71171-y

**Published:** 2020-09-02

**Authors:** Samantha M. Matta, Zachery Moore, Frederick Rohan Walker, Elisa L. Hill-Yardin, Peter J. Crack

**Affiliations:** 1 Department of Pharmacology and Therapeutics, The University of Melbourne, Grattan St, Parkville, VIC Australia; 2 School of Health & Biomedical Sciences, RMIT University, 225-245 Clements Drive, Bundoora, VIC Australia; 3 School of Biomedical Sciences and Pharmacy, University of Newcastle, University Drive, Callaghan, NSW Australia; 4 Hunter Medical Research Institute, Locked Bag 1000, New Lambton, NSW Australia; 5 Department of Physiology, The University of Melbourne, Grattan St, Parkville, VIC Australia

**Keywords:** Neuroscience, Cellular neuroscience

## Abstract

Autism Spectrum Disorder (ASD; autism) is a neurodevelopmental disorder characterised by deficits in social communication, and restricted and/or repetitive behaviours. While the precise pathophysiologies are unclear, increasing evidence supports a role for dysregulated neuroinflammation in the brain with potential effects on synapse function. Here, we studied characteristics of microglia and astrocytes in the Neuroligin-3 (NL3^R451C^) mouse model of autism since these cell types are involved in regulating both immune and synapse function. We observed increased microglial density in the dentate gyrus (DG) of NL3^R451C^ mice without morphological differences. In contrast, WT and NL3^R451C^ mice had similar astrocyte density but astrocyte branch length, the number of branch points, as well as cell radius and area were reduced in the DG of NL3^R451C^ mice. Because retraction of astrocytic processes has been linked to altered synaptic transmission and dendrite formation, we assessed for regional changes in pre- and postsynaptic protein expression in the cortex, striatum and cerebellum in NL3^R451C^ mice. NL3^R451C^ mice showed increased striatal postsynaptic density 95 (PSD-95) protein levels and decreased cortical expression of synaptosomal-associated protein 25 (SNAP-25). These changes could contribute to dysregulated neurotransmission and cognition deficits previously reported in these mice.

## Introduction

Autism Spectrum Disorder (ASD; autism) is a neurodevelopmental disorder estimated to affect 1 in 54 children^1^. Autism is characterised by deficits in social communication, and restricted and/or repetitive patterns of behaviour^2^. A complex interaction between genetic and environmental factors is thought to contribute to autism. The clinical heterogeneity and variability in presentation and severity of autism has made diagnosis, treatment and the study of autism-relevant neurobiology challenging^3^. However, functional and neuroanatomical abnormalities are consistently reported in autism patients^4–6^, with increasing evidence supporting a role for neuroinflammation in autism pathophysiology^7–9^.

Neuroinflammation involves the sustained, and often unwarranted, increase in activity of glial cells (i.e. microglia and astrocytes), which release many pro-inflammatory cytokines and chemokines in response to injury, infection or disease. During this activity, ‘reactive’ microglia and astrocytes show altered morphology. Aberrant neuroimmune profiles have been documented in autism including increased densities of reactive microglia and astrocytes in several brain regions^10–12^ accompanied by alterations in cytokine and chemokine secretion in brain tissue^10,13^, cerebrospinal fluid^10,14^ and blood^15–17^ of autism patients compared to neurotypical controls. In addition to regulating immune function, microglia and astrocytes assist in maintaining synaptic function. Abnormal glial function may therefore influence synaptic circuitry and neuronal connectivity within the central nervous system (CNS) and contribute to regional-specific under-connectivity^6,18–21^ and hyper-connectivity^21,22^ reported in autism. Given the role of glia in regulating synaptic activity, a sustained presence of reactive glial cells could contribute to cognitive and core behavioural traits in autism. It is unclear, however, whether changes to the neuronal architecture cause increased neuroinflammation, or if abnormalities in microglia and astrocytes contribute to aberrant synaptic pruning or dysfunction.

Many mutations in genes encoding synaptic proteins are implicated in autism^23,24^ including a missense mutation whereby an arginine residue is replaced by cysteine at position 451 of exon 7 of the gene encoding neuroligin-3 (NL3). The R451C mutation in NL3 was identified in two brothers diagnosed with autism^25^ and is a strong candidate gene for autism. When expressed in mice (NL3^R451C^ mice), the mutation confers behaviours relevant to the core features of autism including deficits in social interaction^26–29^, reduced vocalisations^30^ and repetitive behaviours^26,31^. Several studies have demonstrated persistent phenotypic traits in NL3^R451C^ mice bred on a mixed and C57/Bl6 background^26,27,29,32^. The R451C mutation causes a reduction in NL3 protein expression at the postsynaptic membrane to approximately 10% of control levels^27,29^. NL3^R451C^ mice also show altered expression of other synaptic scaffolding proteins such as decreased levels of neuroligin 1 (NL1)^29^ and increased expression of postsynaptic density protein 95 (PSD-95) and synapse-associated protein-102 (SAP-102)^27^. These changes occur alongside an imbalance in excitatory and inhibitory synaptic transmission^27,29,33–35^.

Here, we hypothesised that NL3^R451C^ mice exhibit altered microglial and astrocyte morphology indicative of a reactive state as well as changes in synaptic protein levels. We first assessed for changes in neuroinflammation by investigating the density and morphology of hippocampal microglia and astrocytes in WT and NL3^R451C^ mice. We then explored alterations to synapse structure through analysis of synaptic proteins involved in neuronal signalling.

## Results

Microglial density was increased in the DG region of NL3^R451C^ mice compared to WT. In the CA1, microglia somata were elongated in NL3^R451C^ compared to WT mice but did not exhibit any other morphology changes. Astrocyte density was also similar in the hippocampal CA1 and DG regions in NL3^R451C^ and WT mice. Although astrocyte morphological parameters were unchanged in the CA1 region, in the DG, astrocytic branch length, the number of branches, cell radius and cell area measures were decreased in NL3^R451C^ mice. Interestingly, postsynaptic density 95 (PSD-95) protein levels were elevated in the striatum, whereas cortical levels of the presynaptic protein, synaptosomal-associated protein 25 (SNAP-25) were decreased in NL3^R451C^ mice.

### Increased hippocampal microglial density in NL3^R451C^ mice

**Figure 1:**
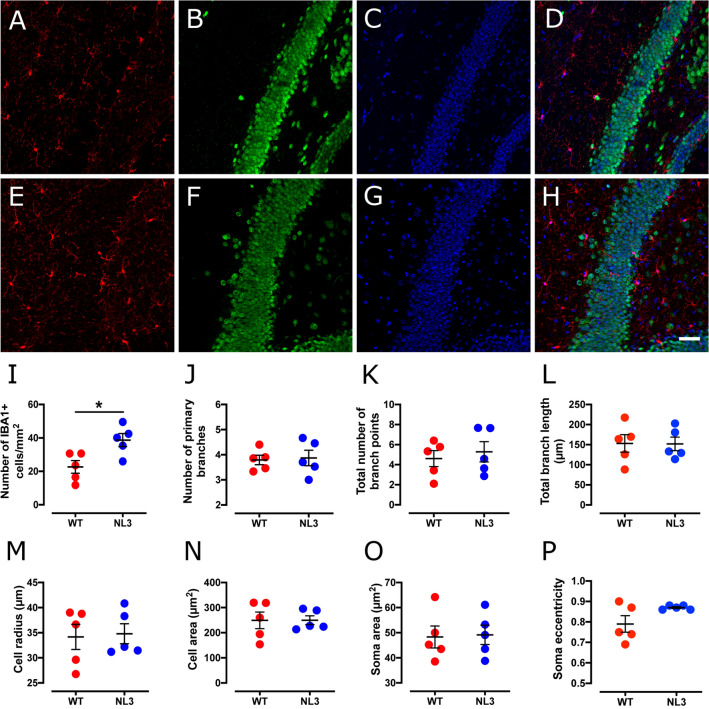
Microglial cell density is increased in the DG hippocampal region in NL3^R451C^ mice. Representative confocal immunofluorescence images of DG hippocampal coronal sections from WT (**A**–**D**) and NL3^R451C^ (**E**–**H**) mice co-labelled with IBA-1 (red), NeuN (green) and DAPI (blue). Scale bar = 50 µm. (**I**–**P**) Number of IBA-1 immunoreactive cells, branching parameters, cell radius, cell area, soma area and soma eccentricity in WT and NL3^R451C^ mice (n = 5 mice in each group). Data represented as mean ± SEM.

**Figure 2:**
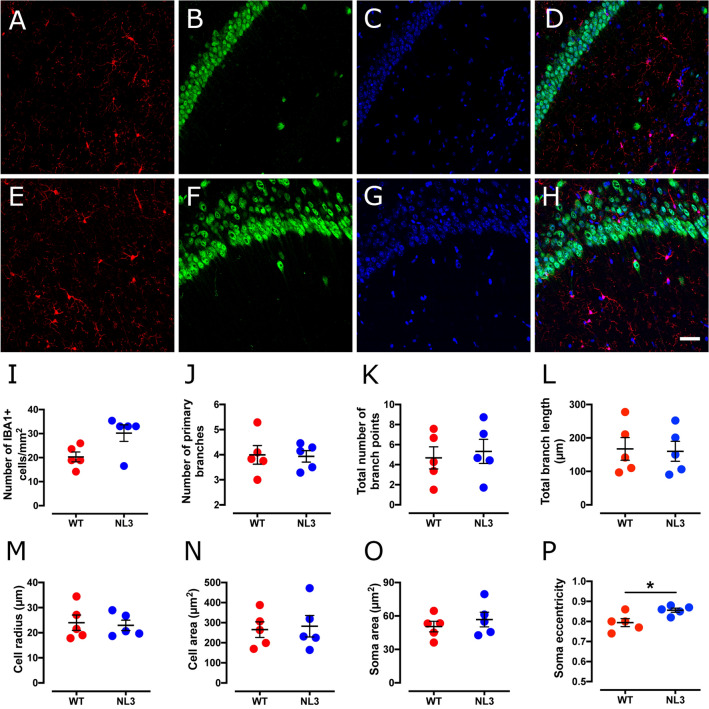
Soma eccentricity is increased in the CA1 hippocampal region in NL3^R451C^ mice. Representative confocal immunofluorescence images of CA1 hippocampal coronal sections from WT (**A**–**D**) and NL3^R451C^ (**E**–**H**) mice co-labelled with IBA-1 (red), NeuN (green) and DAPI (blue). Scale bar = 50 µm. (**I-P**) Number of IBA-1 immunoreactive cells, branching parameters, cell radius, cell area, soma area and soma eccentricity in WT and NL3^R451C^ mice (n = 5 mice in each group). Data represented as mean ± SEM; *p =  < 0.05.

We observed increased microglial density in the DG region (Fig. 1) of the hippocampus in NL3^R451C^ mice compared to WT (Table 1). There was also a non-significant trend for increased density of microglia in the CA1 hippocampal region (Fig. 2). Microglial cell morphology was similar between genotypes with no changes in branching (number of primary branches, number of branch points, or total branch length) or cell size (cell radius or cell area). Although soma area was similar between genotypes, soma eccentricity was increased in the CA1 region of NL3^R451C^ mice.

**Table 1:** Analysis of microglial density and morphology in the hippocampus.

Cell parameters	WT mean	NL3 mean	p-value	Significance
**Hippocampal DG region**				
Cell density (cells/mm^2^)	22.6 ± 4	38.7 ± 4	0.018	*
No. of primary branches	3.79 ± 0.2	3.87 ± 0.3	0.83	NS
No. of branch points	4.61 ± 0.8	5.28 ± 1.0	0.62	NS
Total branch length (µm)	153 ± 21.8	152 ± 16.9	0.97	NS
Cell radius (µm)	34.2 ± 2.5	34.8 ± 2.0	0.85	NS
Cell area (µm^2^)	249 + 33.2	250 ± 17.5	0.99	NS
Soma area (µm^2^)	48.3 ± 4	49.2 ± 4	0.89	NS
Soma eccentricity	0.79 ± 0.04	0.87 ± 0.004	0.08	NS
**Hippocampal CA1 region**				
Cell density (cells/mm^2^)	20.28 ± 2.1	30.19 ± 3.5	0.064	NS
No. of primary branches	3.99 ± 0.4	3.94 ± 0.2	0.90	NS
No. of branch points	4.68 ± 1.1	5.32 ± 1.2	0.70	NS
Total branch length (µm)	167 ± 34	160 ± 30	0.87	NS
Cell radius (µm)	24.0 ± 3	22.9 ± 2	0.79	NS
Cell area (µm^2^)	265.0 ± 39	282.0 ± 53	0.80	NS
Soma area (µm^2^)	50.5 ± 5	56.7 ± 7	0.46	NS
Soma eccentricity	0.79 ± 0.02	0.86 ± 0.01	0.024	*

*NS* not significant.

*p < 0.05.

.

### Reduced cell size and branch length in Dentate Gyrus astrocytes in NL3^R451C^ mice

**Figure 3:**
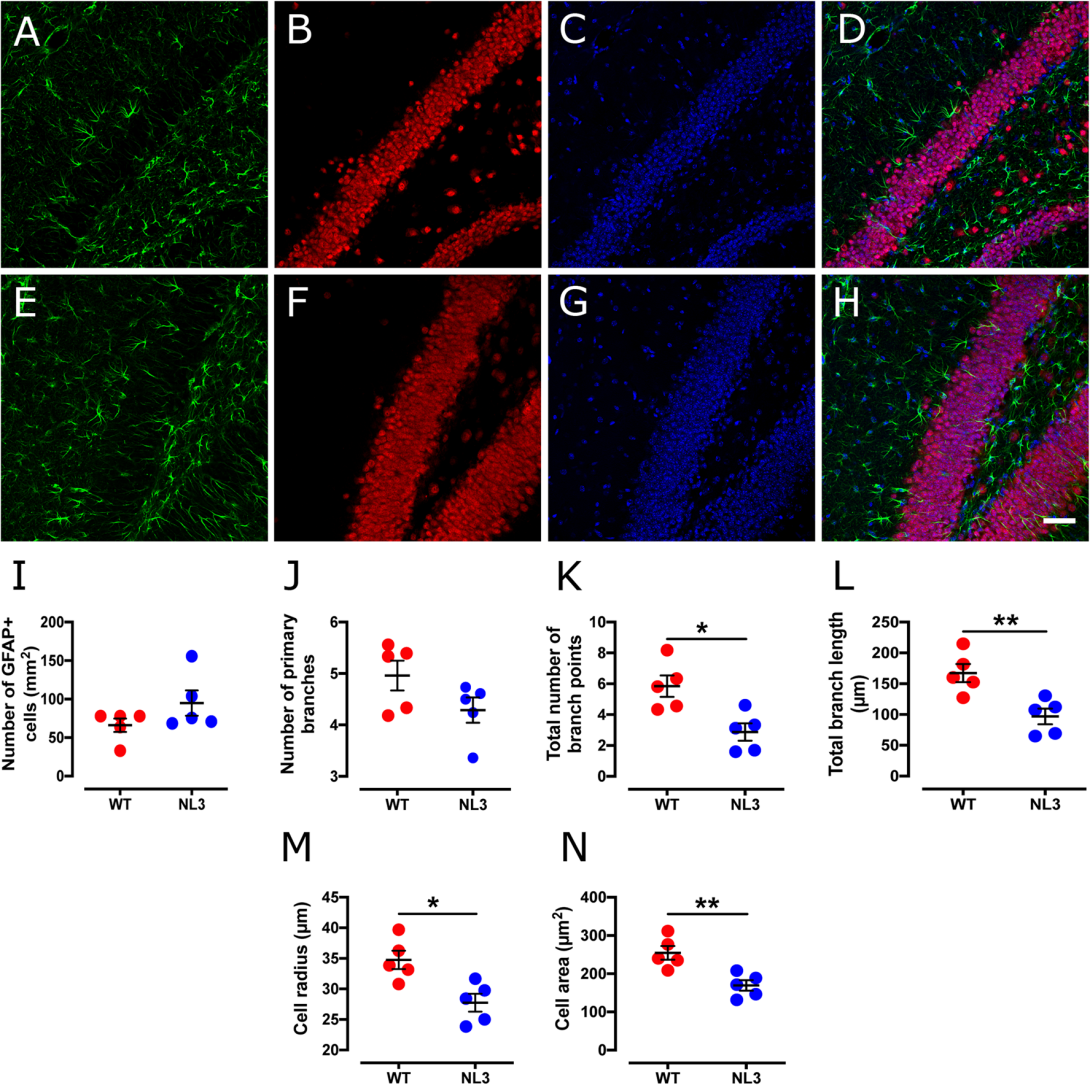
Astrocytic cell density is unchanged, but show lessened number of branch points, retracted processes and decreases in cell radius and cell area in the DG of the hippocampus in NL3^R451C^ mice. Representative confocal immunofluorescence images of DG hippocampal coronal sections from WT (**A**–**D**) and NL3^R451C^ (**E**–**H**) mice co-labelled with GFAP (green), NeuN (red) and DAPI (blue). Scale bar = 50 µm. (**I**–**P**) Number of GFAP immunoreactive cells, branching parameters, cell radius and GFAP labelled cell area (n = 5 mice in each group). Data represented as mean ± SEM; *p =  < 0.05, **p =  < 0.01.

**Figure 4:**
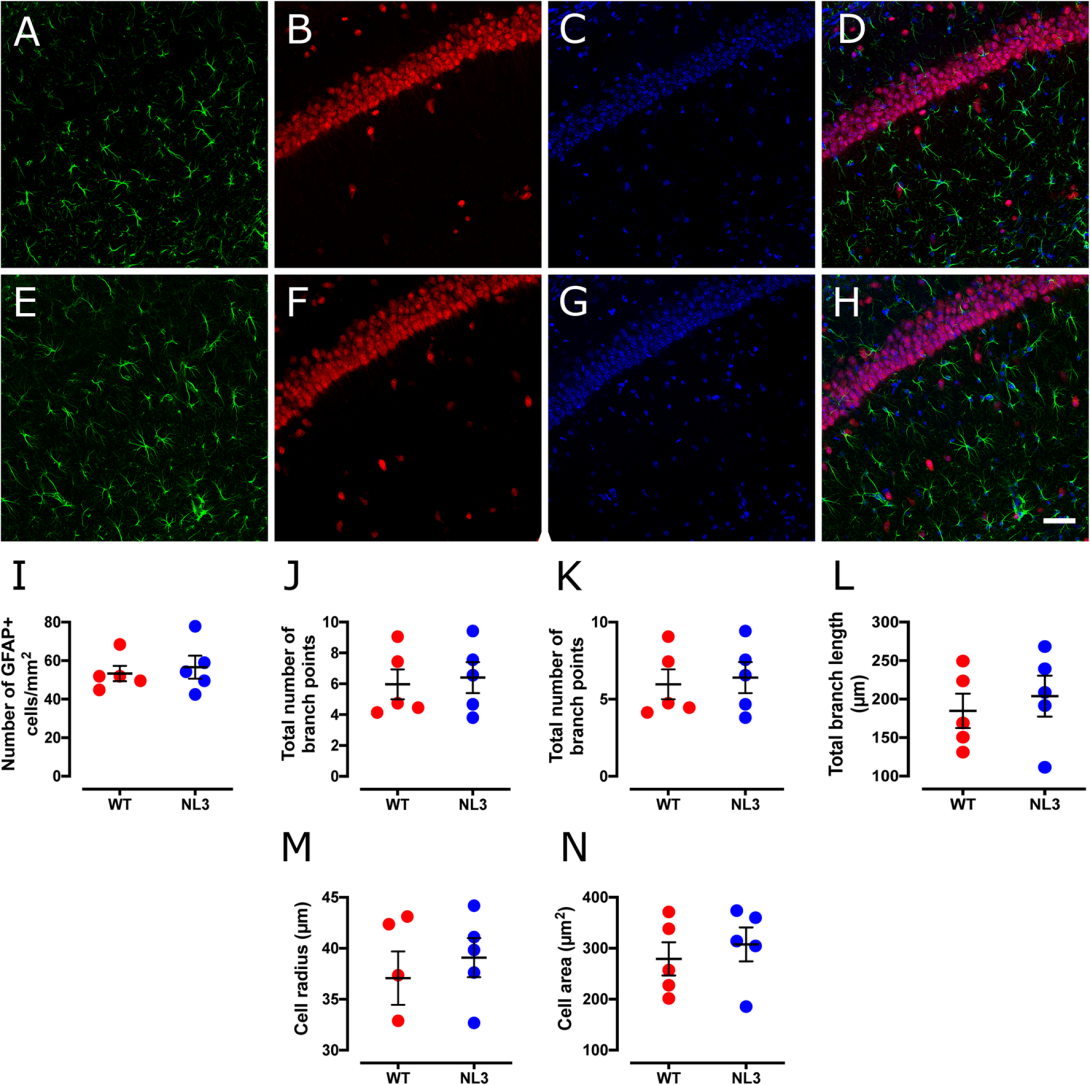
Astrocytic cell density and morphology are unchanged in the CA1 hippocampal region in NL3^R451C^ mice. Representative confocal immunofluorescence images of CA1 hippocampal coronal sections from WT (**A**–**D**) and NL3^R451C^ (**E**–**H**) mice co-labelled with GFAP (green), NeuN (red) and DAPI (blue). Scale bar represents 50 µm. (**I**–**P**) Number of GFAP immunoreactive cells, branching parameters, cell radius and GFAP labelled cell area (n = 5 mice in each group). Data represented as mean ± SEM.

Hippocampal DG (Fig. 3) and CA1 (Fig. 4) astrocytes were present at similar cell densities in WT and NL3^R451C^ mice (Table 2). DG astrocytes, however, showed an altered morphology, with decreased number of branch points, total branch length, cell radius and cell area in NL3^R451C^ mice compared to WT. The total number of primary branches of DG astrocytes was not changed in NL3^R451C^ mice. Astrocyte cellular parameters were similar between NL3^R451C^ and WT mice in the CA1 region.

**Table 2:** Analysis of astrocyte density and morphology in the hippocampus.

Cell parameters	WT mean	NL3 mean	p-value	Significance
**Hippocampal DG region**				
Cell density (cells/mm^2^)	66.1 ± 9	94.8 ± 16	0.16	NS
No. of primary branches	4.96 ± 0.3	4.29 ± 0.2	0.12	NS
No. of branch points	5.84 ± 0.7	2.87 ± 0.6	0.01	*
Total branch length (µm)	167 ± 15	96.8 ± 13	0.007	**
Cell radius (µm)	34.8 ± 1.5	22.7 ± 1.5	0.01	*
Cell area (µm^2^)	254.0 ± 18	169.0 ± 14	0.005	**
**Hippocampal CA1 region**				
Cell density (cells/mm^2^)	53.3 ± 4.0	56.6 ± 6.0	0.72	NS
No. of primary branches	5.79 ± 0.3	5.62 ± 0.3	0.69	NS
No. of branch points	5.96 ± 1.0	6.40 ± 1.0	0.76	NS
Total branch length (µm)	185.0 ± 22	204.0 ± 27	0.60	NS
Cell radius (µm)	37.1 ± 3	39.1 ± 2	0.55	NS
Cell area (µm^2^)	279.0 ± 33	308.0 ± 33	0.56	NS

*NS* not significant.

*p < 0.05, **p < 0.01.

### Region-specific changes in synaptic structural proteins in NL3^R451C^ mice

**Figure 5:**
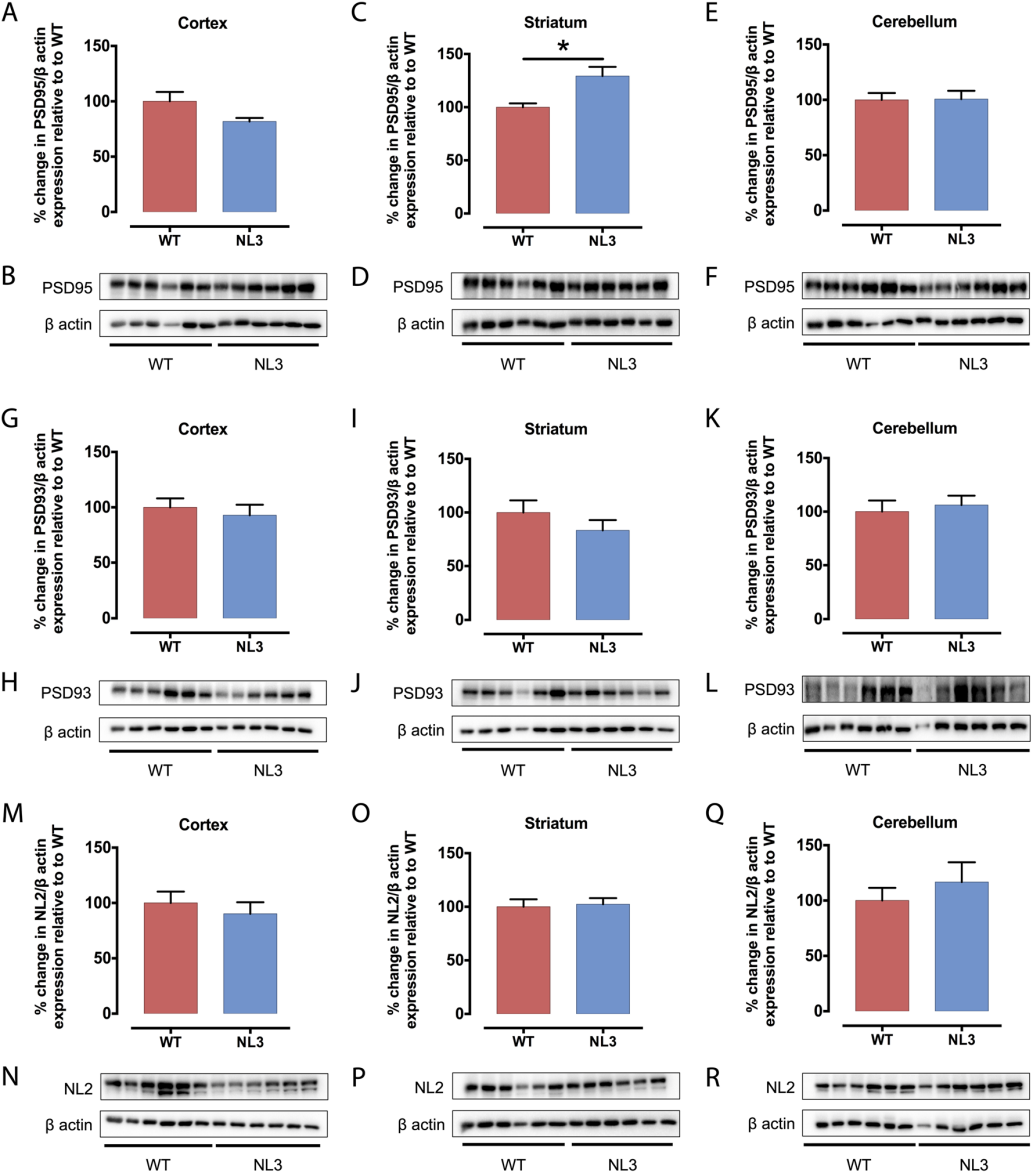
PSD-95 protein levels are increased in the striatum of NL3^R451C^ mice, whereas PSD-93 and NL2 expression is unchanged. Levels of postsynaptic proteins, PSD-95, PSD-93 and NL2 in cortical (**A**,**G**,**M**), striatal (**C**,**I**,**O**) and cerebellar (**E**,**K**,**Q**) brain lysates from NL3^R451C^ and WT mice were analysed via Western blot. Densitometric analysis was performed to demonstrate quantitative expression of PSD-95 (**B**,**D**,**F**), PSD-93 (**H**,**J**,**L**) and NL2 (**N**,**P**,**R**) relative to β actin expression. Genotype differences were analysed using an unpaired, two-tailed Student’s t-test (n = 6 mice in each group); *p < 0.05. Data represented as mean ± SEM.

**Figure 6:**
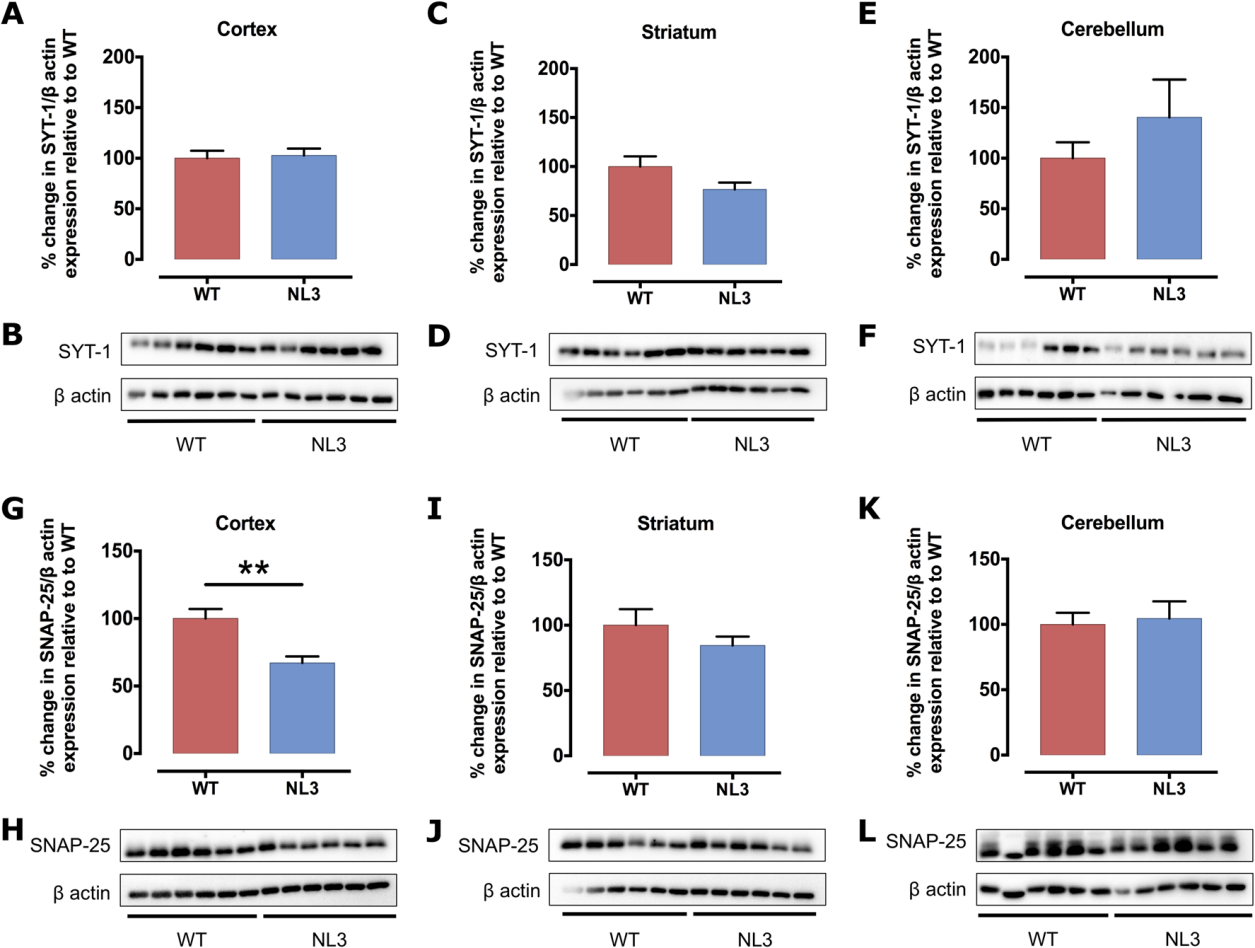
SYT-1 protein levels are unchanged, but SNAP-25 protein expression was decreased in the cortex of NL3^R451C^ mice. SYT-1 and SNAP-25 protein expression in cortical (**A**,**G**), striatal (**C**,**I**) and cerebellar (**E**,**K**) lysates from NL3^R451C^ and WT mice were analysed via Western blot. Densitometric analysis was performed to demonstrate quantitative expression of SYT-1 (**B**,D,**F**) and SNAP-25 (**H**,**J**,**L**) relative to β actin expression. Genotype differences were analysed using an unpaired, two-tailed Student’s t-test (n = 6 mice in each group); **p < 0.01. Data represented as mean ± SEM.

**Figure 7:**
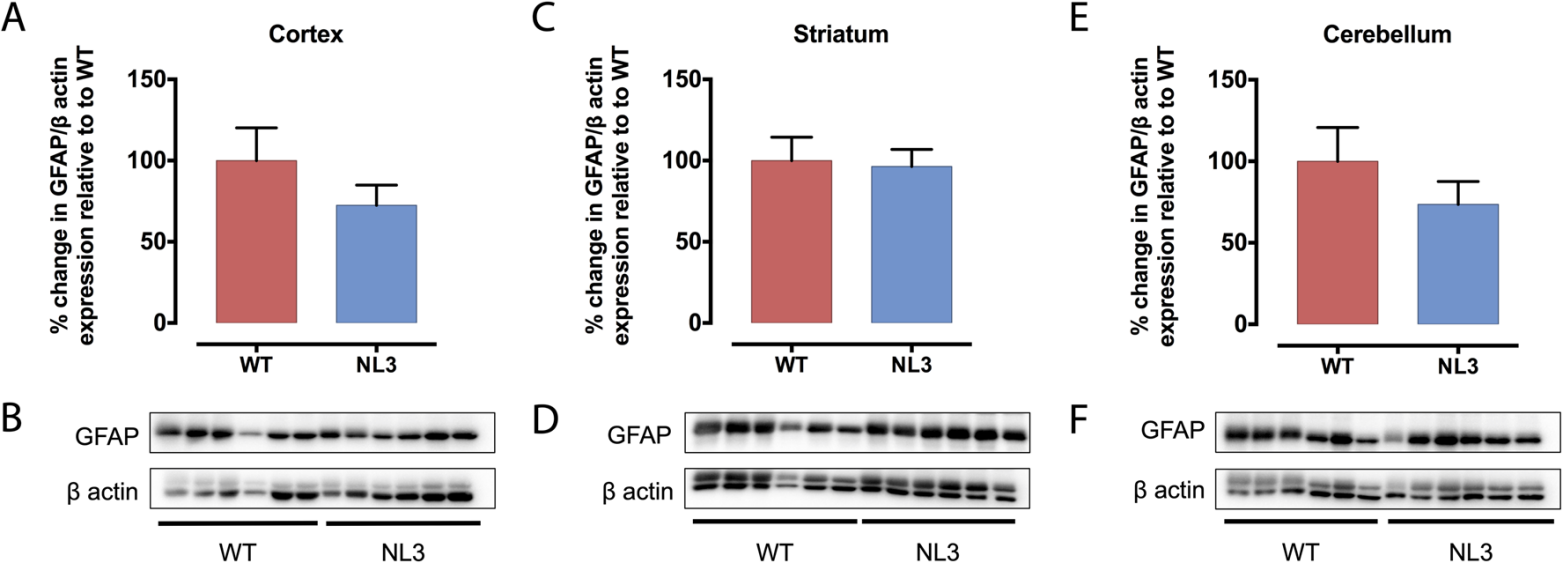
GFAP protein levels are unchanged in NL3^R451C^ mice. Cortical (**A**), striatal (**C**) and cerebellar (**E**) lysates from NL3^R451C^ and WT mice were analysed via Western blot. Densitometric analysis was performed to demonstrate quantitative expression of GFAP relative to β actin expression (**B**,**D**,**F**). Genotype differences were analysed using an unpaired, two-tailed Student’s t-test (n = 6 mice in each group). Data represented as mean ± SEM.

We next investigated the structure of synapses by analysing expression levels of presynaptic (i.e. dendritic; PSD-95, PSD-93 and Neuroligin 2 (NL2); Fig. 5) and postsynaptic (axonal; SNAP-25 and synaptotagmin-1 (SYT-1); Fig. 6) proteins in cortical, striatal and cerebellar tissue samples of WT and NL3^R451C^ mice (Table 3). PSD-95 was increased significantly in the striatum (Fig. 5C,D) and SNAP-25 was decreased significantly in the cortex (Fig. 6G,H) of NL3^R451C^ mice compared to WT. As expected, Neuroligin 3 protein expression was dramatically decreased in all regions analysed in NL3^R451C^ mice (Supplementary Fig. S5). Moreover, in agreement with our GFAP immunofluorescence findings of similar hippocampal astrocyte density in WT and NL3^R451C^ brain slices, we observed no change in GFAP expression levels in WT and NL3^R451C^ cortical, striatal or cerebellar brain lysates (Fig. 7) using Western blot. The original, full-length images of the Western blots have been added to our supplementary information, as Figures S6–S9.

**Table 3:** Analysis of synaptic protein expression.

Synaptic protein	Expression in NL3^R451C^ mice compared to WT		
	Cortex	Striatum	Cerebellum
PSD-95	81 ± 3.2% *p* = *0.071*	129 ± 8.6% p = 0.01*	101 ± 7.6% *p* = *0.95*
PSD-93	93 ± 9.5% *p* = *0.58*	83 ± 9.7% *p* = *0.28*	106 ± 8.8% *p* = *0.66*
NL2	90 ± 10% *p* = *0.52*	102 ± 5.7% *p* = *0.80*	117 ± 18% *p* = *0.45*
SYT-1	103 ± 6.8% *p* = *0.79*	77 ± 6.9% *p* = *0.092*	160 ± 23% *p* = *0.12*
SNAP-25	67 ± 4.8% p = 0.003**	85 ± 6.8% *p* = *0.28*	105 ± 13% *p* = *0.78*
GFAP	72 ± 12% *p* = *0.27*	96 ± 11% *p* = *0.84*	74 ± 14% *p* = *0.32*

The bold, italics font highlights statistically significant differences between genotypes: *p < 0.05, **p < 0.01.

## Discussion

Microglia and astrocyte profiles are subtly altered in NL3^R451C^ mice. These changes differ from previously reported morphological changes thought to indicate a reactive microglial state^36,37^. Namely, microglia in NL3^R451C^ mice did not show process retraction or thickening of ramified processes. In addition, astrocytes did not show hypertrophy or branch extension typically associated with a reactive inflammatory state^36,37^. Interestingly, we observed increased microglial cell density in the DG, and a trend for increased density in the CA1 region, of the hippocampus in NL3^R451C^ mice. Although increased microglial density is typically seen in conjunction with increased reactivity^11,38^, increased microglial density has also been reported prior to the reactive state in a mouse model of Alzheimer’s disease^39^. Rodriguez and colleagues reported an increased density of ramified microglia in the CA1 region of the hippocampus preceding the formation of amyloid β plaques and morphological changes of microglia to a reactive phenotype in a triple-transgenic model of Alzheimer’s disease^39^. Although it is well established that reactive microglia show soma elongation, in the CA1 region of NL3^R451C^ mice we report increased microglial soma eccentricity but no change in other parameters relevant to the reactive state such as soma area, cell size or branching patterns. The microglial soma elongation in NL3^R451C^ mice may indicate a subtle physiological functional change in this cell type, however the precise role of this morphological change in the absence of an inflammatory insult is unclear.

In the DG, we identified a significant decrease in both the number of branch points and the length of astrocyte processes in NL3^R451C^ mutants compared to WT mice. Similarly, significant decreases in measures of cell radius and cell area in DG astrocytes were observed. This may indicate an alteration in the local environment which leads to a subsequent change in cell morphology. Surprisingly, these findings contrast with the morphological changes characteristic of reactive astrocytes, such as an extension and thickening of processes and somatic hypertrophy. Astrocytic processes maintain contact with neuronal synapses and regulate neuronal function as part of the tripartite synapse^40^. Astrocytes also contribute to regulating neurotransmitter concentrations at the synapse through the uptake of the excitatory neurotransmitter glutamate via excitatory amino acid transporters expressed on the astrocytic cell membrane. Prolonged retraction of astrocytic processes could therefore reduce glutamate uptake from the synaptic cleft and alter neurotransmission^41^. Moreover, astrocytic processes interacting with the synapse can prevent synapse growth during memory consolidation, highlighting the significance of astrocyte morphology in synapse function and cognition^42^. For example, astrocyte contact with dendrites regulates the formation, length and survival of dendritic spine protrusions, via pathways involving Ras-related C3 botulinum toxin substrate 1 (Rac1) and ephrin-A3^43^. In addition, retraction and disengagement of astrocytic processes from postsynaptic dendritic spines can decrease the survival rate and maturation of dendrites^44^. As such, our observations could therefore be associated with the formation of immature dendritic spines and disrupted synaptic communication as well as the enhanced motor^26,31^ and spatial learning^28,29^ seen in this model. Relevant to our findings, an increase in dendritic branching but decreased spine area has also been reported in the CA1 region of the hippocampus in NL3^R451C^ mice^27^.

Glial cells are involved in the physiological maintenance of synapses, including the removal, or pruning, of dendrites and axons following prolonged inactivity. Therefore, we next investigated the expression of synaptic structural proteins to determine if synaptic pruning may be altered in this model. Although we found that expression of the majority of synaptic proteins examined were unaffected, regional-specific alterations in expression of cortical SNAP-25 and striatal PSD-95 support previous reports of changes in synapse structure in NL3^R451C^ mice.

Decreased expression of NL1 has previously been reported in NL3^R451C^ mice^29^, but we observed no difference in neuroligin 2 (NL2) protein expression. These observations may be due to differences in the synaptic localisation and function of NL1 and NL2 and their interactions with NL3. NL1 is localised to glutamatergic excitatory postsynapses^45^ whereas NL2 is found at GABAergic inhibitory postsynaptic boutons^46^. NL3 forms heterodimers with NL1 but not NL2^47^. Since the R451C mutation causes a drastic reduction in NL3 protein expression, we propose that decreased NL1 levels may occur in NL3^R451C^ mice due to the reduced levels of NL3 available to bind with NL1.

The postsynaptic density proteins (PSD) PSD-93 and PSD-95 influence neurotransmission via their roles as scaffolding proteins at excitatory synapses as well as by binding NMDA receptors and recruiting AMPA receptors^48^. PSD-95 also binds to neuroligins to facilitate the recruitment of ion channels and receptors to the synapse^49,50^. NL3^R451C^ mice show increased expression of PSD-95 in the hippocampus^27^. In the current study, we confirm these findings via our analysis of PSD-95 protein levels in striatal brain tissue, comprising the hippocampus in addition to the thalamus, amygdala and corpus callosum. Here, we report that elevated PSD-95 protein expression is exclusive to the striatum, as we observed no significant changes in PSD-95 abundance in the cortex or cerebellum despite the expected dramatic decrease in NL3 in all brain regions analysed. Our observations of increased microglial density in the hippocampus may contribute to dysregulation of synaptic maintenance in NL3^R451C^ mice.

Our data show brain region-specific changes in synaptic protein expression in NL3^R451C^ mutant mice. We assayed expression levels of presynaptic proteins, SYT-1 and SNAP-25, which are involved in facilitating neurotransmitter release^51^ and mutations in these genes have been identified in autism patients^52^. Individuals with missense variants in SYT-1 are reported to exhibit impaired social development, motor stereotypies and developmental delay^53^. A number of studies have also reported single nucleotide polymorphisms in SNAP-25 that are associated with increased severity of core behaviours and hyperactivity^54,55^, and are associated with cognitive deficits^56^ in autism. Although SYT-1 abundance was unchanged, SNAP-25 protein levels were significantly decreased in the cortex of NL3^R451C^ mice. SNAP-25 expression has not been previously investigated in this model to our knowledge. Beyond its role in the presynaptic initiation of neuronal signalling and association with autism-relevant behaviours, SNAP-25 influences dendritic spine formation via binding to the postsynaptic protein, p140Cap^57^. Reduced expression of SNAP-25 results in immature dendritic spine formation, specifically involving a decrease in the number of shorter, wider spines, and an increased proportion of longer filopodia^57^. As reported by Tomasoni et al.^57^, these changes were accompanied by a decrease in PSD-95 density and rescued following over-expression of SNAP-25. These findings are relevant given that microglial processes engulf pre- and postsynaptic proteins during synapse maturation in mice. Highlighting this interaction, both PSD-95 and SNAP-25 have been localised to microglia in the CA1 hippocampal region of mice at postnatal day 15^58^. In our analysis, a reduction in cortical SNAP-25 levels was accompanied by a trend for reduced PSD-95 expression in NL3^R451C^ mice, consistent with a potential increase in microglial engulfment of cortical synapses and altered dendrite spine morphology^27^.

This study is the first to identify an increased density of DG hippocampal microglia and decreased expression of cortical SNAP-25 in the NL3^R451C^ mouse model of autism. These initial findings suggest a potential role for microglia, PSD-95 and SNAP-25 in the pathophysiology observed in NL3^R451C^ mice. Although the microglial and astrocytic profiles observed here differ from those associated with immune responses as reported in other rodent models, these subtle changes may predispose NL3^R451C^ mice to an aberrant response in the context of immune stimuli. Furthermore, this phenotype could indicate a role for microglia and astrocytes in altered synapse function in NL3^R451C^ mice. Due to its multi-faceted roles in regulating presynaptic function and postsynaptic dendritic structure, SNAP-25 is a potential candidate target for personalized therapeutic strategies in the treatment of ASD. Collectively, we identified changes to astrocyte morphology that could be involved in mediating previously reported changes in synaptic transmission and dendritic branching in NL3^R451C^ mice^34^. Further characterisation of neuroimmune function in NL3^R451C^ mice is required to advance the understanding of biological mechanisms contributing to autism.

## Methods

### Animals

Immunohistochemical and western blot analyses were performed on tissue from NL3^R451C^ and WT mice. Mice were bred on a Sv129/ImJ/C57Bl6 or a pure C57/Bl6 genetic background. B6; 129-Nlgn3^tm1Sud^/J mice were obtained from Jackson Laboratories (USA) and maintained on a Sv129/ImJ/C57Bl6 background. C57/Bl6 mice were generated after backcrossing onto a pure C57/Bl6 strain for more than 10 generations. Mutant mice express the R451C substitution (arginine to cysteine on residue 451) in exon 7 of the X-linked neuroligin 3 (NL3) gene. Females heterozygous for the NL3^R451C^ mutation were mated with WT males to generate WT and NL3^R451C^ offspring. Only male mice were utilised in these experiments due to the higher prevalence of ASD in males. Mice were housed in mixed genotype groups of up to five per cage on a 12:12 h light/dark cycle, with food and water provided ad libitum. Tail and ear clips were obtained pre-weaning at 3 weeks for genotyping. Genotyping was performed in-house via PCR analysis as previously described^29^. All experiments were approved by the University of Melbourne Animal Ethics Committee (Ethics ID: 1613990.1) and were conducted in line with the Australian code of practice for the care and use of animals for scientific purposes. C57/Bl6 WT and NL3^R451C^ mice used for Western blot analysis were collected at 3 months of age and killed via cervical dislocation (n = 6 for each genotype). Mixed background WT and NL3^R451C^ mice used for immunohistochemical analysis aged 2 to 5 months old were anaesthetised and underwent cardiac perfusion with 4% paraformaldehyde (n = 5 for each genotype, detailed below).

### Immunohistochemistry

Mice were anesthetised via intraperitoneal injection of ketamine (100 mg/kg) and xylazine (10 mg/kg) and perfused transcardially with phosphate buffered saline (PBS; pH 7.4) followed by 4% paraformaldehyde in PBS. The brain was excised and post-fixed for a further 24 h at 4 °C. Brain tissue was subsequently rinsed three times in phosphate buffered saline (PBS) and cryopreserved in 30% sucrose in PBS for at least 24 h at 4 °C. Brains were embedded in optimal cutting temperature (OCT) compound in 2 cm^3^ aluminium foil moulds, submerged partially in isopentane and frozen in liquid nitrogen to prepare for cryosectioning. Moulds were stored at − 80 °C until use.

Coronal sections (30 µm thickness) were cut on a cryostat machine (Reichert-Jung, USA) at − 23 °C until the hippocampal region was reached at bregma -1.955 mm (Allen Reference Atlas). Tissue sections from WT and NL3^R451C^ mice were stained with either the microglial marker, IBA-1 (Ionized calcium-Binding Adapter molecule-1) or the astrocyte marker, GFAP (Glial Fibrillary Acidic Protein) and co-labelled with neuronal marker, NeuN (Neuronal Nuclei) to detect glial cells and neurons in the hippocampus (Table 4). DAPI was used as a counterstain to clarify the cellular organisation of each brain region.

**Table 4:** Antibodies used in immunohistochemical analysis.

Primary antiserum	Supplier	Dilution	Secondary antiserum	Supplier	Dilution
Rabbit polyclonal anti-GFAP	Dako #Z0334	1:500	Goat anti-rabbit IgG Alexa Fluor 488	Invitrogen #A-11008	1:2000
Rabbit polyclonal anti-IBA-1	Novachem #01919741	1:200	Goat anti-rabbit IgG Alexa Fluor 594	Invitrogen #A-11012	1:2000
Mouse monoclonal anti-NeuN	Abcam #ab104225	1:2000	For GFAP: Goat anti-mouse IgG Alexa Fluor 594	Invitrogen #A-11005	1:2000
			For IBA-1: Goat anti-mouse IgG Alexa Fluor 488	Invitrogen #A-11001	1:2000

Free-floating sections were washed (3 × 5 min) and blocked for 1.5 h (for IBA-1) or 3 h (for GFAP) in 1.5% Triton goat block (1 ml goat serum; 9 ml PBS; 150 µl Triton-X (Sigma-Aldrich, Germany)) with constant rocking. Sections were washed (3 × 5 min) and incubated with primary antibodies overnight at 4 °C with constant rocking before being washed (3 × 5 min) and incubated with the corresponding secondary antibodies for 1.5 h in a dark environment with constant rocking. Sections were mounted onto Menzel-Glaser SuperFrost Plus (Thermo Scientific, USA) or regular (Sail Brand, China) microscope slides with Vectashield hard-set mounting medium containing 4′, 5-diamidino-2-phenylindole (DAPI) (Abacus ALS, Australia). Multi-channel images of the DG and CA1 hippocampal regions were taken at 20 × magnification (tissue area imaged was 424 μm^2^) on an LSM880 Airyscan microscope (Zeiss, Germany) at the Biological Optical Microscopy Platform (BOMP), The University of Melbourne, Australia.

### Cell counts and morphological analysis

Cell counts and morphological analysis of microglia and astrocytes was performed using a proprietary method within MATLAB v12.0 as described previously^59,60^. Briefly, the analysis generates intensity quantiles across an image to locate individual cell somas, traces cell processes outwards, then quantifies the resultant image (Supplementary Figs. S1–S4). Minimum object detection (i.e. soma) size was set at 100–200 pixels. Quantile levels for both soma and background intensity were manually adjusted in order to normalise fluorescence intensity between tissue samples. The resulting optimal intensity level enabled clear detection of signal intensities in contrast with low levels of background labelling. The entire field of view (424 µm^2^) of the regions of interest (hippocampal DG and CA1) were counted at × 20 magnification.

### Western blot analysis

Mice were killed via cervical dislocation prior to decapitation and brains excised. Brain tissue from the left hemisphere was divided in cortical, striatal and cerebellar tissue samples and for Western blot analysis. Tissue was suspended in 900 µl of homogenisation buffer (9.88 ml Milli-Q H_2_O , 125 µl 1 M Tris HCl pH 7.4, 2.5 ml 10% sodium dodecyl sulfate (SDS), 1 tablet PhosSTOP phosphatase inhibitor (Roche, Switzerland), 1 tablet cOmplete protease inhibitor (Roche)) using a homogenising pestle and sonication (15 × 1 s pulses). Protein samples were gently rotated in a rotary suspension mixer at 4 °C for 1.5 h. Samples were centrifuged (Eppendorf Centrifuge 5424 R, Germany; 15 min at 12,000 rpm) and the colourless supernatant extracted. A Bradford Protein Assay (BioRad, USA) was performed to determine the total protein content of the brain lysates as per manufacturer’s instructions. Protein samples were diluted in sample buffer (5% β-mercaptoethanol (v/v) (Sigma-Aldrich) in 2 × Tris–Glycine SDS loading buffer (Invitrogen, USA)) to achieve a final concentration of 2 µg/µl. Samples were heated at 95 °C for 15 min to denature the proteins and stored at -20 °C until use.

10 µl of brain lysate samples (for a final protein concentration of 20 µg/µl) and 5 µl of the pre-stained molecular weight ladder (BioRad) (8% gel: 25—250 kDa; 12% gel: 10—70 kDa) were loaded on an 8% resolving gel (per four gels: 10 ml 1.5 M Tris–HCl (BioRad), 400 µl 10% (w/v) SDS (BioRad), 18.98 ml Milli-Q H_2_O, 10.67 ml 30% Acrylamide/Bis solution (BioRad), 600 µl 10% (w/v) Ammonium Persulfate (APS, Sigma-Aldrich) in Milli-Q H2O, 60 µl N,N,N′,N′-tetramethylethane-1,2-diamine (TEMED, BioRad)) or 12% resolving gel (per four gels: 10 ml 1.5 M Tris–HCl, 400 µl 10% (w/v) SDS, 13.16 ml Milli-Q H_2_O, 16 ml 30% Acrylamide/Bis solution, 600 µl 10% APS in Milli-Q H_2_O, 40 µl TEMED). Proteins were separated by SDS-PAGE at 80/120 V in Tris–Glycine SDS-PAGE running buffer (Life Technologies, USA). The separated sample proteins in the gel were transferred onto a Polyvinylidene fluoride (PVDF) membrane via a semi-dry transfer which was run at 60 mA/gel for 75 min, facilitated by a transfer buffer (20 ml of 25 × Tris–glycine transfer buffer (Life Technologies), 50 ml 100% methanol, 430 ml Milli-Q H_2_O). The PVDF membrane was then blocked in 5% (w/v) skim milk in Tris Buffer Solution Tween 20 (TBS-T; 5% Tween-20 (v/v) in TBS) for 1 h at RT with constant rotation. Membranes were washed (3 × 10 min) and incubated in the primary antiserum diluted in TBS-T (Table 5) overnight at 4 °C. The membrane was washed (3 × 10 min) then incubated in the corresponding horseradish peroxidase (HRP)-conjugated secondary antiserum diluted in TBS-T for 1.5 h at RT.

**Table 5:** Antibodies used in Western blot analysis.

Primary antiserum	Supplier	Dilution	Secondary antiserum	Supplier	Dilution
Rabbit polyclonal anti-NL3 (110 kDa)	Synaptic systems #129113	1:500	Goat anti-rabbit IgG HRP	Dako #P0048	1:1,000
Rabbit polyclonal anti-PSD-93 (110 kDa)	Alomone Labs #APZ002	1:500			
Rabbit polyclonal anti-NL2 (91 kDa)	Synaptic systems #129203	1:500			
Rabbit polyclonal anti-SYT-1 (48 kDa)	Alomone Labs #ANR-003	1:500			
Rabbit polyclonal anti-GFAP (48 kDa)	Dako #Z0334	1:5,000			
Rabbit polyclonal anti-SNAP-25 (23 kDa)	Alomone Labs #ANR-001	1:500			
Mouse monoclonal anti-PSD-95 (95 kDa)	UC Davis/NIH NeuroMab Facility #K28/43	1:5,000	Goat anti-mouse IgG HRP	Dako #P0447	1:1,000
Mouse monoclonal anti-β-actin (42 kDa)	Sigma-Aldrich #A5441	1:4,000			

Membranes were imaged using a ChemiDoc XRS + system (BioRad) and protein bands were visualised using the electrochemiluminescence (ECL) prime detection kit (Amersham, USA). To determine whether the total protein content was constant across samples, β-actin was used as a loading control. Optical density of the bands was analysed via densitometric analysis using ImageJ software (version 2.0.0-rc-68/1.52e; NIH, USA), and arbitrary intensity units were compared by calculating the expression of the antiserum of interest relative to the loading control (β-actin), and averaged for both the WT and NL3^R451C^ genotypes. Mean protein expression values for WT mice were normalized to 100% for each blot and NL3^R451C^ protein expression levels were compared as a percentage relative to control (WT).

### Statistical analysis

Statistical analysis was performed using Prism software (Graphpad v6.0c; USA). All cell datasets were assessed for normality with the Kolmogorov–Smirnov test for normality with the Dallal-Wilkinson-Lilliefor correction. For datasets that passed the normality test, potential differences between samples from the two genotypes were analysed using a two-tailed, unpaired Student’s t-test. Datasets that failed to meet a normal distribution (CA1 microglial density and CA1 astrocyte density) were assessed using a two-tailed Mann–Whitney U test. For Western blot analysis, all expression levels in NL3^R451C^ mutant tissue were compared to WT levels (normalised to 100) and analysed with a two-tailed unpaired Student’s t-test. Data was presented as mean ± SEM; p-values less than 0.05 were considered to represent statistical differences between genotypes.

## Supplementary information

Supplementary file1
