# Supplementary material for: An altered glial phenotype in the NL3R451C mouse model of autism
Source: Sci Rep. 2020 Sep 2;10:14492. doi: 10.1038/s41598-020-71171-y (PMC7468159; doi:10.1038/s41598-020-71171-y)
Supplement: Supplementary file 1 — Supplementary file1 [file 41598_2020_71171_MOESM1_ESM.docx]

**Supplementary information**

**An altered glial phenotype in the NL3^R451C^ mouse model of autism**

Samantha M. Matta^1,2^, Zachery Moore^1^, Frederick Rohan Walker^4,5^,

Elisa L. Hill-Yardin^2,3^, Peter J. Crack^1*^

^1^ Department of Pharmacology and Therapeutics, The University of Melbourne, Grattan St, Parkville, VIC, Australia

^2^ School of Health & Biosciences, RMIT University, 225-245 Clements Drive, Bundoora, VIC, Australia

^3^ Department of Physiology, The University of Melbourne, Grattan St, Parkville, VIC, Australia

^4^ School of Biomedical Sciences and Pharmacy, University of Newcastle, University Drive, Callaghan, NSW, Australia

^5^ Hunter Medical Research Institute, Locked Bag 1000, New Lambton, NSW, Australia

*Corresponding author: pcrack@unimelb.edu.au


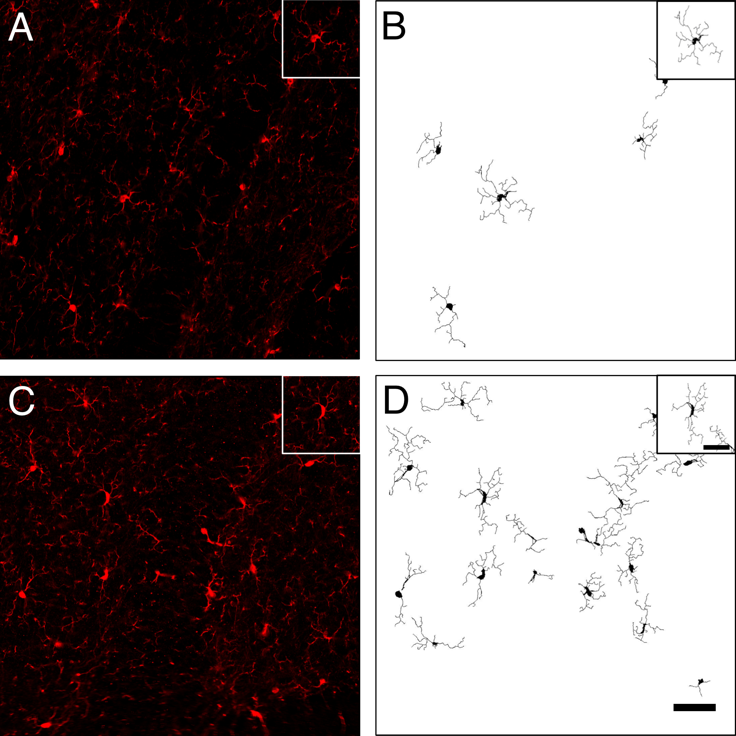


**Supplementary Figure S1**

Representative images of DG hippocampal microglial cells and respective cell traces are shown in (**A, B**) WT and (**C, D**) NL3^R451C^ mice. Scale bar, main panel = 50 μm. Scale bar, insert = 30 µm.


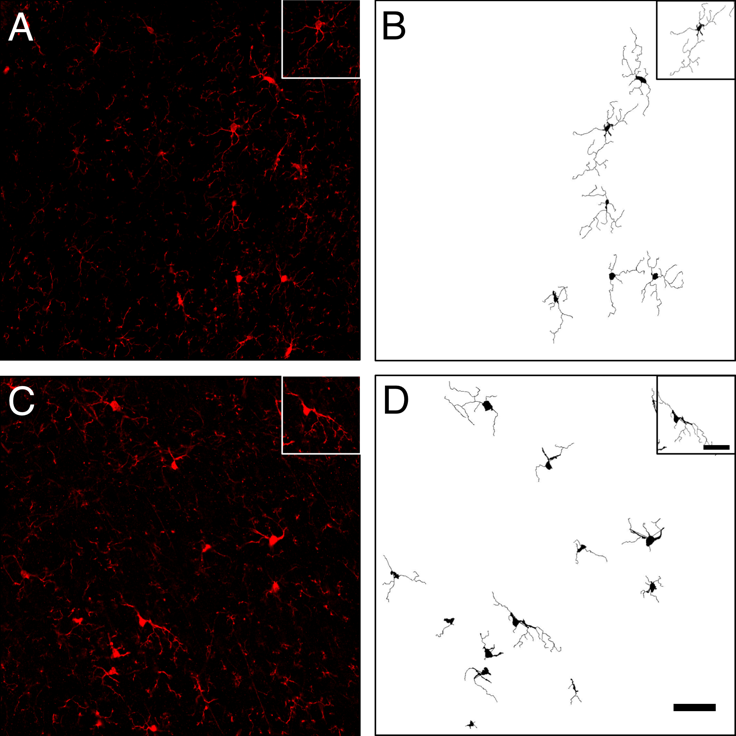


**Supplementary Figure S2**

Representative images of CA1 hippocampal microglial cells and respective cell traces are shown in (**A, B**) WT and (**C, D**) NL3^R451C^ mice. Scale bar, main panel = 50 μm. Scale bar, insert = 30 µm.


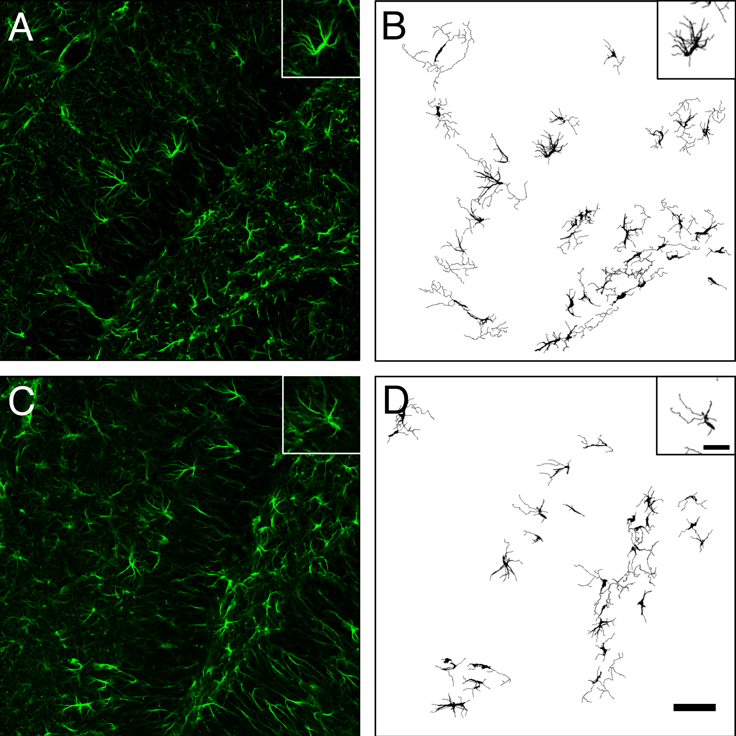


**Supplementary Figure S3**

Representative images of DG hippocampal astrocyte cells and respective cell traces are shown in (**A, B**) WT and (**C, D**) NL3^R451C^ mice. Scale bar, main panel = 50 μm. Scale bar, insert = 30 µm.

**
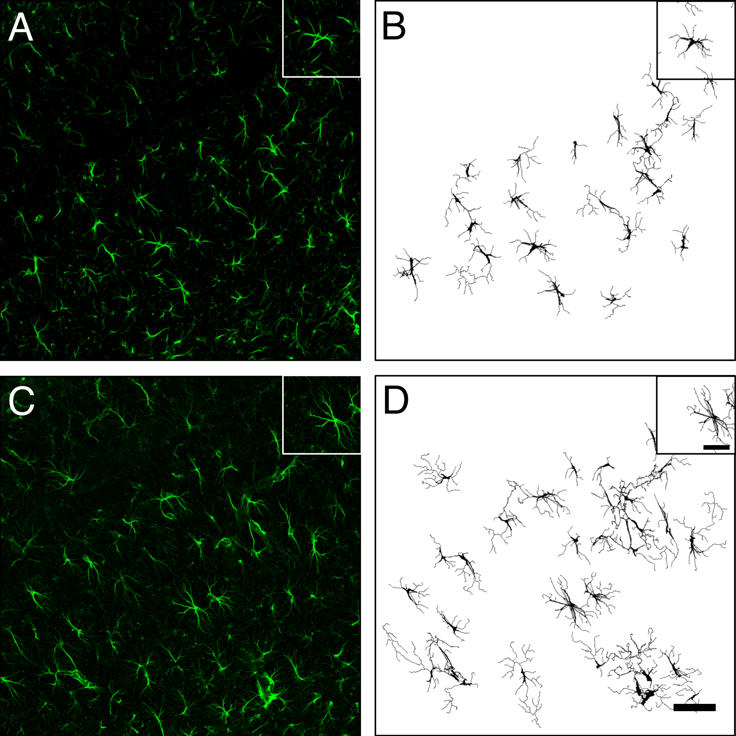
**

**Supplementary Figure S4**

Representative images of CA1 hippocampal astrocyte cells and respective cell traces are shown in (**A, B**) WT and (**C, D**) NL3^R451C^ mice. Scale bar, main panel = 50 μm. Scale bar, insert = 30 µm.


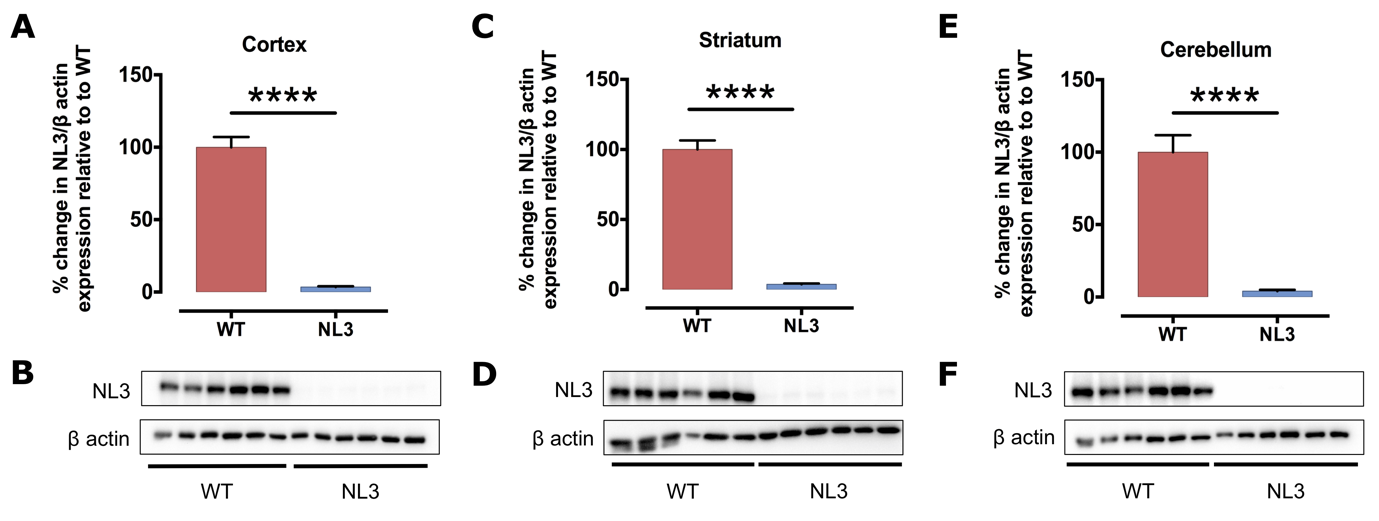


**Supplementary Figure S5**

NL3 protein levels are decreased in NL3^R451C^ mice. Cortical (**A**), striatal (**C**) and cerebellar (**E**) lysates from NL3^R451C^ and WT mice were analysed via Western blot. Densitometric analysis was performed to demonstrate quantitative expression of NL3 relative to β actin expression (**B, D, F**). Genotype differences were analysed using an unpaired, two-tailed Student’s t-test (n = 6 mice in each group); ***p < 0.001. Data represented as mean ± SEM.


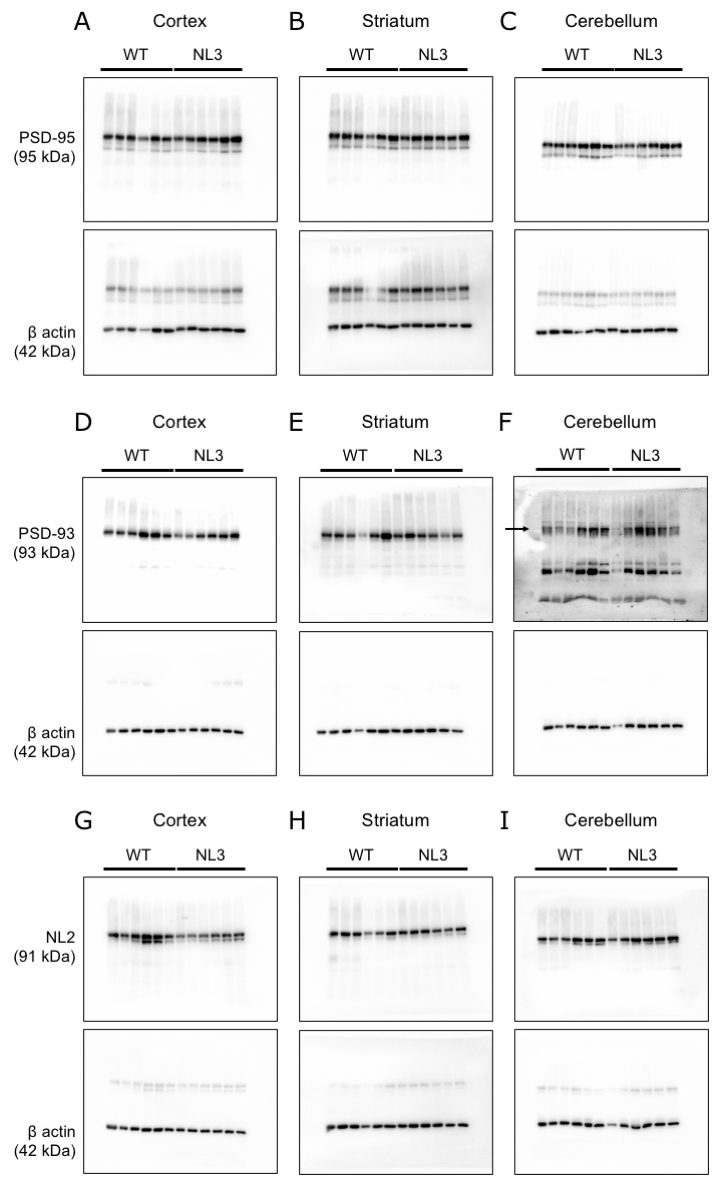


**Supplementary Figure S6**

Original, full length images of the Western blots for cortical, striatal and cerebellar expression of PSD-95 (**A-C**), PSD-93 (**D-F**) and NL2 (**G-I**) in NL3^R451C^ and WT mice.


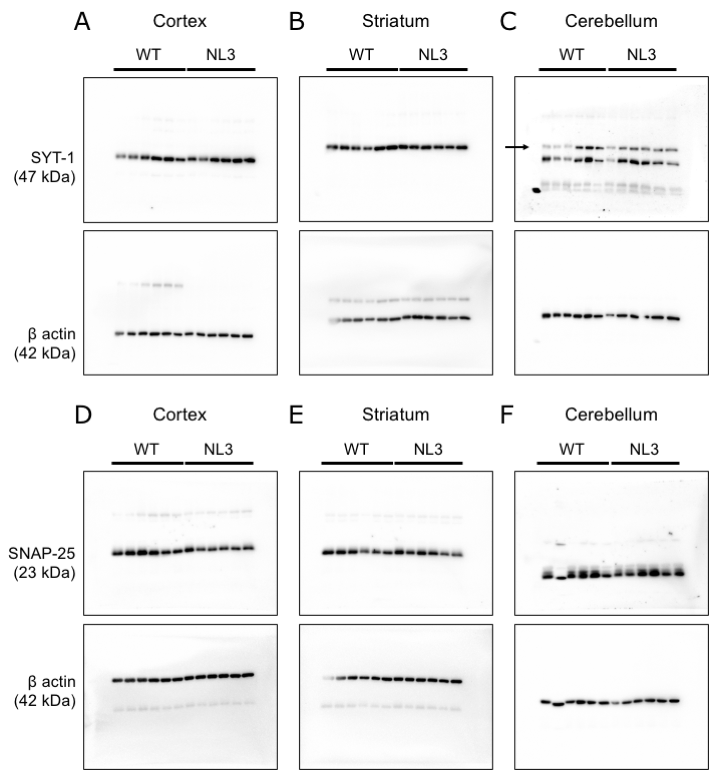


**Supplementary Figure S7**

Original, full length images of the Western blots for cortical, striatal and cerebellar expression of SYT-1 (**A-C**) and SNAP-25 (**D-F**) in NL3^R451C^ and WT mice.


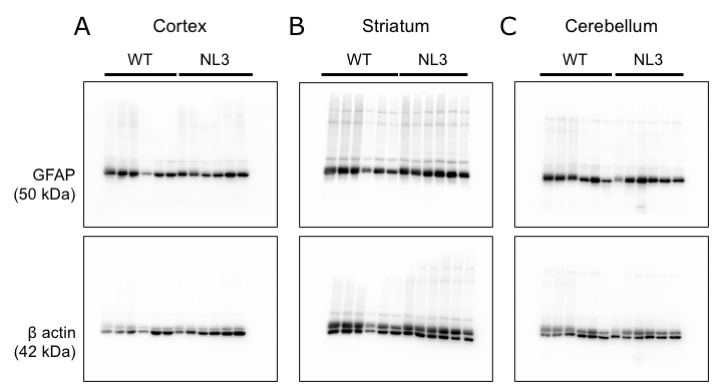


**Supplementary Figure S8**

Original, full length images of the Western blots for cortical, striatal and cerebellar expression of GFAP (**A-C**) in NL3^R451C^ and WT mice.


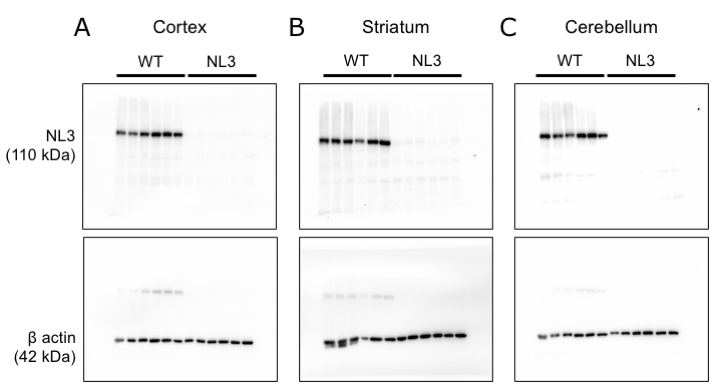


**Supplementary Figure S9**

Original, full length images of the Western blots for cortical, striatal and cerebellar expression of NL3 (**A-C**) in NL3^R451C^ and WT mice.
